# Monitoring peripheral blood data supports the prediction of immunotherapy response in advanced non-small cell lung cancer based on real-world data

**DOI:** 10.1007/s00262-025-03966-9

**Published:** 2025-02-25

**Authors:** Ana D. Ramos-Guerra, Benito Farina, Jaime Rubio Pérez, Anna Vilalta-Lacarra, Jon Zugazagoitia, Germán Peces-Barba, Luis M. Seijo, Luis Paz-Ares, Ignacio Gil-Bazo, Manuel Dómine Gómez, María J. Ledesma-Carbayo

**Affiliations:** 1 Biomedical Image Technologies, Escuela Técnica Superior de Ingenieros de Telecomunicación, Universidad Politécnica de Madrid, Madrid, Spain; 2 Centro de Investigación Biomédica en Red de Bioingeniería, Biomateriales y Nanomedicina, Instituto de Salud Carlos III, Madrid, Spain; 3 Hospital Universitario Fundación Jiménez Díaz, IIS-FJD, Madrid, Spain; 4 Memorial Sloan Kettering Cancer Center, New York, USA; 5 Department of Medical Oncology, Clínica Universidad de Navarra, Pamplona, Spain; 6 Centro de Investigación Biomédica en Red de Cáncer, Instituto de Salud Carlos III, Madrid, Spain; 7 Hospital Universitario 12 de Octubre, Madrid, Spain; 8 Centro de Investigación Biomédica en Red de Enfermedades Respiratorias, Instituto de Salud Carlos III, Madrid, Spain; 9 Department of Oncology, Hospital Vithas Vitoria, Vitoria, Spain; 10 School of Medicine, Universidad Católica de Valencia, Valencia, Spain

**Keywords:** Non-small-cell lung cancer, Peripheral blood biomarker, Immunotherapy, Longitudinal analysis, Prognostic marker, Real-world data

## Abstract

The identification of non-small cell lung cancer (NSCLC) patients who will benefit from immunotherapy remains a clinical challenge. Monitoring real-world data (RWD) in the first cycles of therapy may provide a more accurate representation of response patterns in a real-world setting. We propose a multivariate Bayesian joint model using generalized linear mixed effects, trained and validated on RWD from 424 advanced NSCLC patients retrospectively collected from three clinical centers. Center1 was used as training ($N=212$), while Center2 and Center3 were used as independent testing sets ($N=137$ and $N=75$, respectively). Peripheral blood data (PBD) were collected at baseline and at three follow-up time points, alongside demographic and epidemiologic features. Six models were trained to predict progression-free survival at 6 months, PFS(6), using different number of longitudinal samples (baseline, two, or four time points) of the neutrophil-to-lymphocyte ratio (NLR) or a multivariate feature selection. Long-term predictions at 12 and 24 months were also evaluated. Prediction accuracy was measured using the area under the receiver operating characteristic curve (AUC). The proposed model significantly improved prediction performance, achieving AUCs of 0.870, 0.804 and 0.827 at 6, 12 and 24 months for Center2, and 0.824, 0.822 and 0.667 for Center3. There was also a significant difference in PFS and overall survival (OS) between predicted response groups, defined by a 6-month PFS cutoff (log-rank test $p<0.001$). Our study suggests that the integration of multiple biomarkers and monitored PBD in an RWD-based Bayesian joint model framework significantly improves immunotherapy response prediction in advanced NSCLC compared to conventional approaches involving biomarker data at baseline only.

**Supplementary Information:**

The online version contains supplementary material available at 10.1007/s00262-025-03966-9.

## Introduction

Lung cancer is the leading cause of cancer death worldwide and exhibits a 5-year survival rate of 19% [1]. In case of distant metastasis, which accounts for 57% of all lung cancer patients, the survival rate drops to 5%. The most prevalent diagnosis is non-small cell lung cancer (NSCLC), which represents 85% of all lung cancer cases [1]. In addition to more conventional treatments, such as chemotherapy and radiotherapy, immune checkpoint inhibitors have widened the scope of cancer treatments, significantly improving patient prognosis by enhancing the immune response and reversing the tumor’s immune-blocking mechanisms [2]. Despite pathways underlying immunotherapy response have been thoroughly explored, they do not always provide the proper clinical response even in eligible subjects [3, 4]. Studies indicate that a significant proportion of those who initially respond to first-line therapy eventually experience disease progression, along with the potential risk of severe and life-threatening side effects [5].

Predicting the response to immunotherapy would facilitate the development of optimal treatment strategies, enable the focus of clinical trials on specific patient groups, enhance the effectiveness and personalization of patient care, minimize the probability of unnecessary side effects, and reduce healthcare costs [6]. A growing number of decision support tools have been proposed that make use of a wide range of data sources, including clinical, radiological, histopathological, and genomic information. These tools often exploit the latest advances in statistical, machine learning, and deep learning techniques [7]. However, these studies typically demand large cohorts and interpreting results presents a significant challenge. Furthermore, studies often exclude cases with loss to follow-up, which introduces bias, restricts the pool of available patients and increases the complexity of algorithm training [8].

The utilization of data collected from routine clinical practice, also known as real-world data (RWD), may offer a more comprehensive understanding of cancer survival and progression in a real-world setting [9]. Among the well-established predictive biomarkers derived from blood samples in cancer, the neutrophil-to-lymphocyte ratio (NLR) is regarded as one of the most straightforward standard indicators of systemic inflammation [10]. The predictive value of NLR has been demonstrated at both baseline and over time, although studied in a discrete, rather than longitudinal, manner [11]. Other ratios and scores have been widely studied and are used to assess the general condition of the patient, cancer progression, nutritional status, and the immune and inflammatory tumor context. These include the monocyte-to-lymphocyte ratio (MLR), platelet-to-lymphocyte ratio (PLR), systemic immune-inflammation index (SII), advanced lung cancer inflammation index (ALI) and prognostic nutritional index (PNI) [12–14].

It has been observed that alterations in the tumor-associated immune landscape occur during therapy. Blood tests are typically performed before each immunotherapy cycle, making hemogram results easy to use as a monitoring method. Consequently, the use of pretreatment information as a sole predictor of patient progression may be inadequate, despite its frequent use in previous research [15].

Joint modeling (JM) is a robust statistical approach that accounts for the dependence and association between longitudinal and time-to-event data. Rizopoulos enabled the incorporation of multiple longitudinal variables while ensuring realistic computation times and developed accessible software for JM implementation [16]. The methodology has since been implemented in other software tools such as *Stan, Monolix*, and *NONMEM* [17, 18].

JMs offer several advantages over traditional time-series methods [19], such as accounting for censored data and providing uncertainty quantification, an essential feature when dealing with limited, temporal, and incomplete clinical datasets. JMs also show a reduced bias in parameter estimation and an increased efficiency of statistical inference over other methods [20].

In oncology, JM applications mainly focus on pharmacodynamics and lesion kinetics, as seen in recent research [21, 22]. Very few studies have explored the relationship between longitudinal peripheral blood data beyond NLR and time-to-event data in cancers other than NSCLC [23]. In this context, a survival Bayesian joint model, trained with RWD collected over time, may effectively capture immune changes that occur during the initial cycles of immunotherapy. Such changes may serve to either increase or decrease the risk of progression and death in patients with NSCLC. In this study, we evaluate the performance of the proposed model in comparison with a Cox proportional hazard approach that relies exclusively on baseline and first-cycle information. Furthermore, we assess the efficacy of a multivariate setting in contrast to an NLR univariate approach.

## Materials and methods

### Patient cohort

We conducted a retrospective study of 514 advanced NSCLC patients from three clinical centers: Hospital Universitario Fundación Jiménez Díaz (Center1, $N=219$), Clínica Universidad de Navarra (Center2, $N=174$), and Hospital Universitario 12 de Octubre (Center3, N=121). Patient inclusion criteria incorporated (a) confirmed stage III-IV NSCLC, excluding the neuroendocrine subtype due to its unique treatment response [24]; (b) treatment consisting of immunotherapy as monotherapy or a combination of immune-based agents, or concurrently with conventional (e.g., chemotherapy, radiation therapy) or targeted therapies (TT); (c) availability of baseline peripheral blood data (PBD), clinical and epidemiological information.

**Fig. 1:**
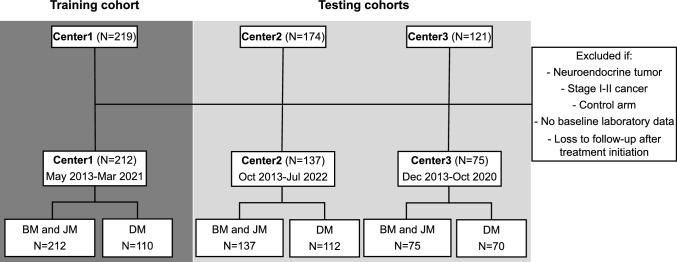
Flowchart of study cohorts. The final number of patients and the follow-up temporal range is provided for the Center1 training set and Center2 and Center3 independent testing sets. Patients who were excluded from the study belonged to the neuroendocrine subtype, NSCLC stage I-II and/or the control arm of an immunotherapy clinical trial. Additionally, patients without baseline hemogram data and/or loss to follow-up after initiating therapy were also not considered. This study analyzed models using (1) baseline data exclusively (BM), (2) the difference between data from the first and second immunotherapy cycle (DM) and (3) any available longitudinal data up to the fifth immunotherapy cycle (JM). Patients lacking second-cycle data were excluded from the DM analysis

Given the inclusion criteria, the final cohorts comprised 424 patients with advanced NSCLC who received immunotherapy treatment in a variety of settings, covering routine clinical care, clinical trials, expanded access, and compassionate use programs (see Fig. 1). Treatment comprised programmed cell death protein 1 (PD-1)/PD-L1 inhibitors (pembrolizumab, atezolizumab, durvalumab, nivolumab and cemiplimab) and other immunotherapy drugs: anti-CTLA-4, anti-ICOS, anti-TIM-3, anti-FAP-IL-2v and anti-CSF1R. The age of the patients varied from 32 to 89 years, including 307 men (72%) and 117 women (28%).

This retrospective study was approved by the institutional review boards of the three clinical centers involved and informed consent was collected accordingly.

### Data collection

PBD was collected from electronical health records (EHRs), covering complete blood cell counts, lactate dehydrogenase (LDH) and albumin (ALB) levels. PBD also comprised multiple scores reported to be potentially predictive and/or prognostic biomarkers for NSCLC: NLR, MLR, PLR, ALI, SII and PNI. Data was collected at baseline and during three follow-up time points within the first 3 months of treatment, corresponding to immunotherapy cycles C1, C2, C3, and C5. The time between administration cycles varied between immunotherapy drugs due to differing regimens and discontinuation due to immune-related adverse effects (irAEs).

Data on demographic profile, surgery, tumor characterization, antibiotic and steroid use, irAEs and other medical complications were also collected. irAEs considered were diarrhea, thyroiditis, pneumonitis, colitis, hepatitis, adrenal insufficiency, dermatitis, hypophisitis, and encephalitis. The variables used to model the progression to immunotherapy are shown in Table S1.

### Response assessment

Progression-free survival (PFS) was defined as the time from the first cycle of immunotherapy to clinical or radiological progression, leading to the end of treatment, death from any cancer-related cause, or last follow-up [25]. RECIST response, categorized as complete response, partial response, stable disease, or progressive disease, along with overall response rate (ORR), was collected to describe the population. Overall survival (OS) was the time from treatment initiation to death from cancer-related cause or last follow-up. All models were trained to predict PFS at 6 months, PFS(6), ensuring a consistent comparison across models and facilitating model evaluation. Patients with a PFS greater than 6 months were considered responders [26]. Inferences were made at 6, 12 and 24 months to evaluate the long-term predictive ability of models.

### Statistical analysis and model design

#### Data split

Center1 (212 patients) was used as training, while the Center2 and Center3 were used as independent testing sets, with 137 and 75 patients, respectively. The training set was further divided into a tuning (70%) and a validation set (30%) for optimal model feature selection, ensuring a balanced representation of responders and non-responders. After tuning, the final model was trained on the entire Center1 dataset.

#### Data preprocessing

One-hot encoding was applied for categorical variables, and missing baseline data was imputed using mean and mode values from the training set. For Center3, variables not collected (ALB, surgical history and pack years) were imputed for all patients. The amount of imputation required per feature and clinical center is described in Table S2. Since only two variables had > 20% of missing at random in the training set, we considered the inclusion of all collected variables for the imputation and further feature selection processes. Longitudinally, not all study variables were collected consistently for each immunotherapy cycle. For each patient, those time points for which a blood test was not performed were excluded. Missing values at a given time point for which hemogram was carried out but did not have complete information were imputed. The methodology used maintains the integrity of PBD evolution profiles by using a different imputation approach for each case [27]: the rolling mean [28], if previous and posterior time points were available; the last observation carried forward [29], if only the previous time point was present; the next observation carried backward [30], if the following recorded value was available; or the mean, if no information was collected.

Continuous variables were normalized using min–max normalization for a more stable optimization process, better balanced gradients, improved covariance estimation, and enhanced convergence in the joint model.

#### Model design

Two different models were trained: Cox proportional hazards model (CPH) and Bayesian joint model (JM) [31]. For JM, a linear mixed-effects model was used as the longitudinal submodel, while a CPH model was used as the survival submodel to facilitate performance comparison with the BM and DM CPH models. In CPH the baseline hazard is left unspecified, providing flexibility and ensuring that the model is not constrained to a particular distribution of survival times. Schoenfeld residuals were analyzed to check if the proportional hazards assumption was met. CPH allowed to study the effect of each model variable on the risk of experiencing progression. JM also modeled the average longitudinal trajectory (slope), the baseline mean value associated to each monitored variable or intercept, and the error ($\sigma$). Given intercepts and slopes in the longitudinal submodel and HRs from the survival submodel, we can identify how temporal changes affect progression in relation to other model features.

For JM models, the default setup in the R package *JMbayes* was used (version 0.8–85). Model parameters were estimated by drawing Markov Chain Monte Carlo (MCMC) samples from the joint posterior distribution given the parameter prior distribution. Multivariate mixed effects were implemented in *JAGS* through *rjags* (version 4.10) and it was assumed that temporal information followed a Gaussian distribution. For priors, we followed the standard assumptions of the *JMbayes* framework. Specifically, independent univariate normal priors were assigned to the regression coefficients in both the longitudinal and survival submodels, as well as to the vector of association parameters. Variance parameters for normal longitudinal outcomes were modeled using inverse-gamma priors, while variance–covariance matrices were assigned an inverse Wishart distribution. Parameter sampling was performed using random walk Metropolis, except for the precision parameter of the error terms, for which slice sampling was used. 10,000 iterations were performed and the burn-in period was set to 3000 iterations. For assessing algorithm convergence, we fitted four separate MCMC chains and computed the Gelman–Rubin diagnostic $\hat{\hbox {R}}$.

#### Feature and model selection

Wilcoxon Mann–Whitney, Pearson ${\chi }^2$ and Fisher’s exact tests were used to compare feature differences between clinical centers. Models differ in the subset of variables and use of monitored features. For multivariate models, a stepwise selection was used, based on the area under the time-dependent ROC curve (AUC) in the validation set. The null CPH AUC was used as reference. A multivariate selection (MV) was compared with using NLR. Three additional model variants were considered: baseline models (BM) using data from C1, delta models (DM) using the difference between data from C1 and C2 (i.e., $\Delta$variable), and longitudinal models (JM) incorporating data from C1, C2, C3, and C5.

In summary, six models were compared: MV-BM, MV-DM, MV-JM, NLR-BM, NLR-DM and NLR-JM. The optimal threshold for categorizing patients as responders (PFS>6) and non-responders (PFS<6) was determined using maximally selected rank statistics via 4-fold cross-validation on the Center1 training set and the average value was applied to the testing sets.

#### Model evaluation

Hazard ratios (HR) were used to analyze the effect and importance of each model variable, where HR<1 indicated lower progression risk and HR>1 indicated higher risk. The HRs computed by the models correspond to a one-unit change in the normalized scale ($\hbox {HR}_{\text {norm}}$) for the normalized continuous variables. For interpretability purposes, we also present the HRs for a one-unit change in the variable’s original scale, $\hbox {HR}_{\text {orig}}$ (see Tables S4–S9). Parameter identifiability was not reached for variables with a relative standard error (RSE) exceeding 50%, based on the investigated data. The 10-fold cross-validation information criterion (10-fold IC) was used to assess the goodness of fit of the different models. Time-dependent AUC, sensitivity, and specificity values were used to analyze the discriminatory power at 6, 12 and 24 months [32]. Youden index (J) identified the model with the best sensitivity–specificity tradeoff. Standard deviations (SD) for all metrics were computed by bootstrapping with 1000 replications. Kaplan–Meier method and log-rank test compared OS and PFS probabilities between predicted responder or non-responder groups, significant if p<0.05. Simulated survival curves were averaged from predicted individual curves. Model evaluation was conducted on the two testing sets to evaluate robustness. Additionally, we computed AUC considering the data from all centers.

All data analysis and model generation was performed using R version 3.6.0.

## Results

### Population characteristics

Significant differences were observed in the outcomes and the immunotherapy regimens assigned for each clinical center (Table 1). First-line immunotherapy was the most common choice for Center1 (131, 62%), with pembrolizumab as the most used therapy (139, 66%), while fewer Center3 patients received first-line treatment (10, 13%) and pembrolizumab (12, 16%). A lower rate was also observed in Center2: 52 (38%) first-line and 54 (39%) pembrolizumab cases. The first-line treatment group exhibited significantly longer PFS and OS (PFS $p<0.001$, OS $p<0.05$) across the entire population. Monotherapy was the only treatment provided to all patients in H120 (75 patients, 100%), while it accounted for 56% of treatments in Center1 (118 patients) and 55% in Center2 (75 patients). No significant differences were observed when comparing monotherapy with combined immunotherapy protocols across the entire population. The distribution of PFS and OS was significantly independent according to the long-rank test for the Center1 and Center3 clinical centers (PFS $p<0.01$, OS $p<0.001$). PFS and OS curves of Center1, Center2 and Center3 clinical centers, line, and treatment option are shown in Fig. S1.

**Table 1:** Summary of patient outcomes and treatment information for individuals from Center1 (train set), and Center2 and Center3 (independent test sets). *p*-value of difference between Center1 and Center2 and Center3 was computed by the Wilcoxon Mann–Whitney test for continuous features and Pearson ${\chi }^2$ or Fisher’s exact test for categorical variables

	Center1	Center2	*p*	Center3	*p*
*No. of patients, n(%)*	212 (50)	137 (32)		75 (18)	
*PFS, months*			0.270		0.05
Median	5.60	3.80		3.30	
95% CI	8.02$-$10.95	8.48$-$13.40		4.98$-$9.10	
*Progression, n(%)*	157 (74)	116 (85)	*	68 (91)	** $^{\text {a}}$
*Progression after 6 mo, n(%)*	100 (47)	58 (42)	0.44	28 (37)	0.18
*ORR, n(%)*	63 (30)	27 (20)	*	13 (17)	0.05
*RECIST response, n(%)*					
Complete response	13 (6)	13 (9)	0.24	1 (1)	0.18
Partial response	50 (24)	14 (10)	**$^{\text {a}}$	12 (16)	0.23
Stable disease	43 (20)	6 (4)	*** $^{\text {a}}$	25 (33)	*
Progressive disease	83 (39)	66 (48)	0.14	37 (49)	0.09
*OS, months*			0.22		0.70
Median	10.00	11.00		12.00	
95% CI	12.18$-$16.19	15.60$-$22.48		10.41$-$14.96	
*Dead, n(%)*	100 (47)	96 (70)	***	62 (83)	*** $^{\text {a}}$
*Line, n(%)*					
1st	131 (62)	52 (38)	*** $^{\text {a}}$	10 (13)	*** $^{\text {a}}$
2nd	56 (26)	48 (35)	0.11	50 (67)	*** $^{\text {a}}$
3rd or more	25 (12)	37 (27)	*** $^{\text {a}}$	15 (20)	0.12
*Treatment, n(%)*					
Monotherapy	118 (56)	75 (55)	0.95	75 (100)	***
Combined IO	12(6)	27 (20)	*** $^{\text {a}}$	0 (0)	0.08
IO & CT	65 (31)	24 (17)	**	0 (0)	*** $^{\text {a}}$
IO & RT	12 (6)	8 (6)	1.00	0 (0)	0.08
IO & TT	5 (2)	3 (2)	1.00	0 (0)	1.00
*Immune drug, n(%)*					
Pembrolizumab	139 (66)	54 (39)	*** $^{\text {a}}$	12 (16)	***
Atezolizumab	23 (11)	33 (24)	** $^{\text {a}}$	7 (23)	*
Nivolumab	13 (6)	46 (34)	*** $^{\text {a}}$	28 (37)	***
Other	37 (17)	4 (3)	*** $^{\text {a}}$	18 (24)	0.29
*PDL-1, n(%)*					
< 1%	50 (24)	28 (20)	0.58	62 (83)	***
1–50%	87 (41)	81 (59)	0.51	6 (8)	***
$\ge$ 50%	75 (35)	28 (20)	**	7 (9)	***

$$^{*}$$ p value<0.05, $$^{**}$$ *p*-value < 0.01, $$^{***}$$ *p*-value < 0.001, $$^{\text {a}}$$Fisher exact test IO: Immunotherapy, IO &CT: IO & Chemotherapy, IO&RT: IO & Radiotherapy, IO&TT: IO & Targeted therapy

Demographic, clinical and PBD data collected from training and testing sets and used as input in models are described in Table S1. Patients in Center3 had significantly less patients with a ECOG-PS score of 0, only 9 (12%), in contrast to the 76 (36%) seen in Center1 and 42 (31%) in Center2. Center3 had significantly more patients with < 1% PDL-1 positive tumor cells, 62 patients (83%), in contrast to the 50 (24%) and 28 (20%) patients observed in Center1 and Center2, respectively. Additional differences were observed in sex, BMI, smoking status, steroid and proton pump inhibitor (PPI) use, histology, driven mutations, number of distant metastases and metastatic sites, and collected PBD levels.

### Baseline and temporal patterns of progression

**Fig. 2:**
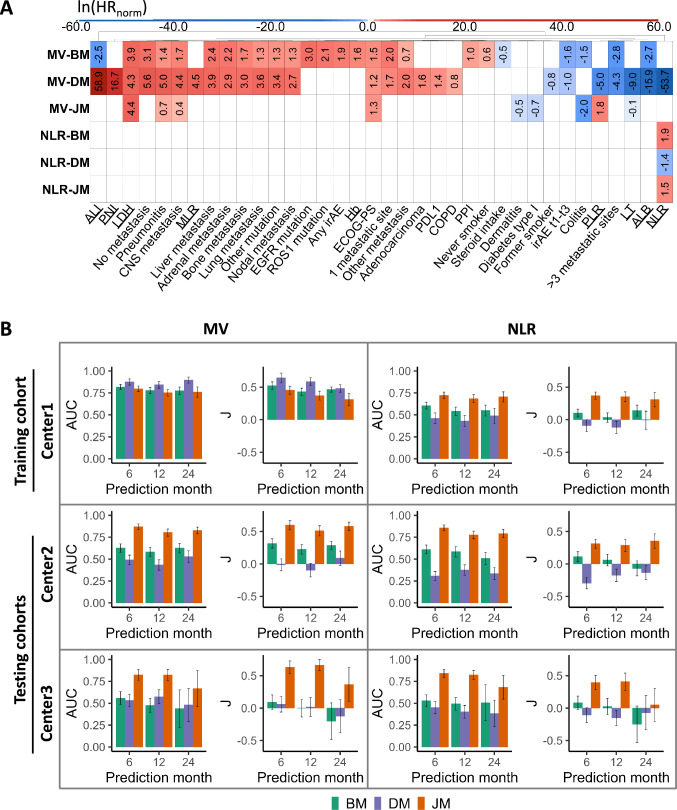
In A, the logarithm of $\hbox {HR}_{\text {norm}}$ computed by each model, in the normalized scale, is displayed. HRs represent the relative risk of progression, taking into account the other features also included in each model. ln($\hbox {HR}_{\text {norm}})>0$ indicates an increased risk of progression at 6 months (red) and ln($\hbox {HR}_{\text {norm}})<0$ shows a lower progression risk (blue). Feature selection was performed independently for each model with excluded features shown in white (ln($\hbox {HR}_{\text {norm}})=0$). The models differ by the selection of variables used (multivariate: MV, univariate: NLR) and the use of monitored variables (baseline: BM, delta: DM, longitudinal: JM). $\hbox {HR}_{\text {norm}}$ for MV-JM and NLR-JM models are associated with the survival submodel of the longitudinal model, and monitored features were further used in the longitudinal submodel for making final predictions. In B, AUC and Youden index (J) results in training set (Center1) and in two independent testing sets (Center2 and Center3) when predicting progression at 6, 12 and 24 months. Standard deviations are shown as error bars. It can be noted that MV-BM and MV-DM performance dramatically drops from training to testing results, showing overfitting

Out of 14 monitored PBD biomarkers and 20 baseline variables, 65 features were generated after converting categorical variables into dummy. For multivariate models, 25 (Table S4) and 26 (Table S6) variables were selected for baseline (MV-BM) and delta (MV-DM) models, respectively. 9 features were selected for the longitudinal approach (MV-JM), as detailed in Table S8. Schoenfeld residual test was significant for MV-BM ($p<0.001$) and MV-DM ($p<0.05$), indicating that the PH assumption is not met for these models and associated with coefficient bias. For both MV-JM and NLR-JM final models, the Gelman–Rubin diagnostic $\hat{\hbox {R}}$ associated with each parameter estimate was equal to 1.00 or less than 1.05, indicating good convergence of the MCMC algorithm. The impact on progression of selected features in each model, expressed as HRs, is shown in Fig. 2A, including NLR-based models with baseline (NLR-BM), delta (NLR-DM) and longitudinal (NLR-JM) data.

Variables in the multivariate and longitudinal MV-JM model take into account the patient’s general condition (ECOG-PS, diabetes type I), tumor progression (CNS metastasis), the immune response triggered (pneumonitis, colitis and dermatitis), and the systemic inflammatory state of cancer (LDH, LT and PLR). Significant differences were found between the training and testing sets for ECOG-PS and baseline LDH (see Table S3). Compared to MV-JM, the multivariate baseline and delta MV-BM and MV-DM models included more features related to metastasis location: liver, kidney, bone, nodal, lung and other. The multivariate and baseline MV-BM model also included information about driver mutations (EGFR, ROS1) and medication (steroids, PPI), together with characteristics related to smoking status, number of metastatic sites, irAEs, ALB, Hb and ALI. The multivariate and delta MV-DM model additionally considered features related to biomarker expression (PD-L1) and tumor histology (adenocarcinoma), in addition to other information about COPD, smoking status, number of metastatic locations, irAEs, $\Delta$ALB, $\Delta$NLR, $\Delta$MLR, $\Delta$PNI, and $\Delta$ALI.

MV-JM modeled trajectories of temporal variables are represented in Fig. S2 along with the trajectories from several responders (red) vs non-responder cases (blue) from the testing sets. The intercepts correspond to the reference value for high or low probability of progression at baseline. For the MV-JM model, a rapid increase in LDH, a rapid decrease in LT, and a very slow decrease or change in PLR resulted in higher probability of progression. Similarly, in the NLR-JM model, a very slow decrease or no change in NLR also predicts a higher chance of progression. Observing the trajectories of the three variables for the responders vs non-responders patients we can verify that they match the temporal patterns defined by the model. Model features, HRs with their p values and RSE values are shown in Tables S4–S9. Most predictors in all 6 models (MV-BM, NLR-BM, MV-DM, NLR-DM, MV-JM and NLR-JM) have low RSEs (< 50%), suggesting good parameter identifiability and indicating that the primary insights from the models are robust.

### Model performance among different progression times and patient groups

Considering the computed probabilities of progression at 6, 12, and 24 months, the multivariate and longitudinal MV-JM model demonstrated a significant performance improvement over the multivariate and NLR-based baseline and delta models in both Center2 and Center3 clinical centers (testing sets): $\hbox {AUC}^{\text {Center2}}_{6\text {mo}}$ = 0.870 (0.031), $\hbox {AUC}^{\text {Center2}}_{12\text {mo}}$ = 0.804 (0.040), $\hbox {AUC}^{\text {Center2}}_{24\text {mo}}$ = 0.827 (0.039) and $\hbox {AUC}^{\text {Center3}}_{6 \text {mo}}$ = 0.824 (0.060), $\hbox {AUC}^{\text {Center3}}_{12 \text {mo}}$ = 0.822 (0.064), $\hbox {AUC}^{\text {Center3}}_{24 \text {mo}}$ = 0.667 (0.206). If combined all three clinical centers: $\hbox {AUC}^{\text {All}}_{6 \text {mo}}$ = 0.832 (0.021), $\hbox {AUC}^{\text {All}}_{12 \text {mo}}$ = 0.787 (0.025), $\hbox {AUC}^{\text {All}}_{24 \text {mo}}$ = 0.792 (0.034). The multivariate baseline and delta MV-BM and MV-DM models showed overfitting in Center1, reflected in the lack of generalization when faced with new cases in Center2 and Center3 (see Fig. 2B). In Table S10, 10-fold IC analysis demonstrates that the multivariate approach consistently outperforms NLR across all three modeling frameworks (BM, DM, JM), as reflected by lower information criterion values.

We investigated potential differences in the AUCs of the multivariate and NLR-based longitudinal models (MV-JM and NLR-JM) for both Center2 and Center3 clinical centers; both longitudinal models obtained higher AUC values ($p<$ 0.05) and lower standard deviations at 6, 12 and 24 months compared to baseline and delta models in these two hospitals. No significant differences were observed between both longitudinal models MV-JM and NLR-JM. AUC at 24 months was slightly lower in Center3, what could be explained by differences in the mean progression-free survival time in this population (Fig. S1). A summary with AUC, sensitivity and specificity of the MV and NLR models is shown in Tables S11 and S12, respectively.

**Fig. 3:**
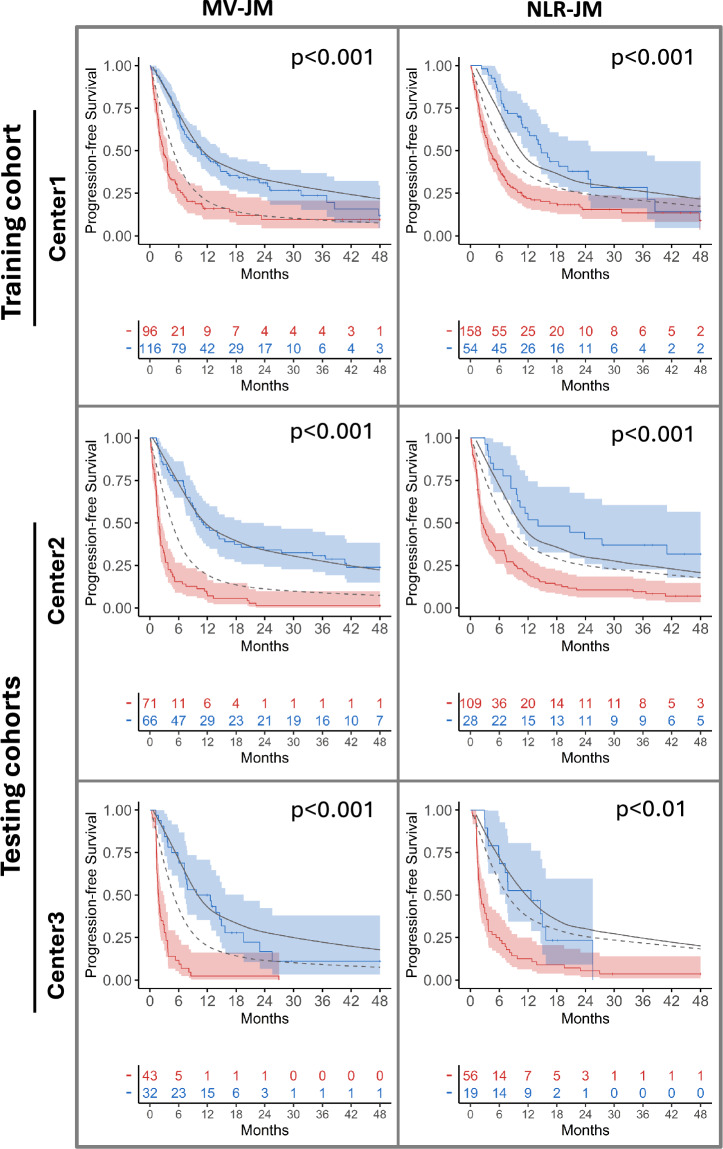
PFS KM curves estimated by the longitudinal and multivariate MV-JM model and the longitudinal and univariate NLR-JM model predicting responder (blue) or non-responders (red) in Center2 and Center3 testing sets. Simulated survival curves are shown in gray, with solid lines representing responders and dashed lines representing the non-responder group. Plotted groups were generated by the corresponding cutoff value for each model, computed in the training set and applied to both testing sets. Log-rank test p-value for KM curves is displayed

When the population was categorized according to the probability of progression after 6 months according to the threshold computed in Center1 (see Figures S3–S5), a low sensitivity or specificity was observed in the multivariate baseline and delta models, and NLR-based baseline, delta and longitudinal models, reflected in poor *J* values (see Fig. 2B and supplementary materials Tables S11 and S12). In this sense, the multivariate and longitudinal MV-JM model achieved significantly more balanced sensitivity and specificity in Center2 and Center3 clinical centers, with significantly higher *J* values. Despite the low specificity observed for the longitudinal NLR-JM model, Kaplan–Meier PFS and OS curves were significantly different for responder and non-responder groups predicted. A higher significancy and narrower confidence interval bands for the multivariate and longitudinal MV-JM model were shown, demonstrating that this model may be a better predictor of progression to immunotherapy in advanced NSCLC. Survival predictions generated by MV-JM align more closely with observed Kaplan–Meier estimates compared to those from NLR-JM. In the case of the Center3 cohort, the simulated curves from MV-JM were less accurate, likely due to limited data availability and variations in treatment protocols (Figs. 3 and Figure S6).

**Fig. 4:**
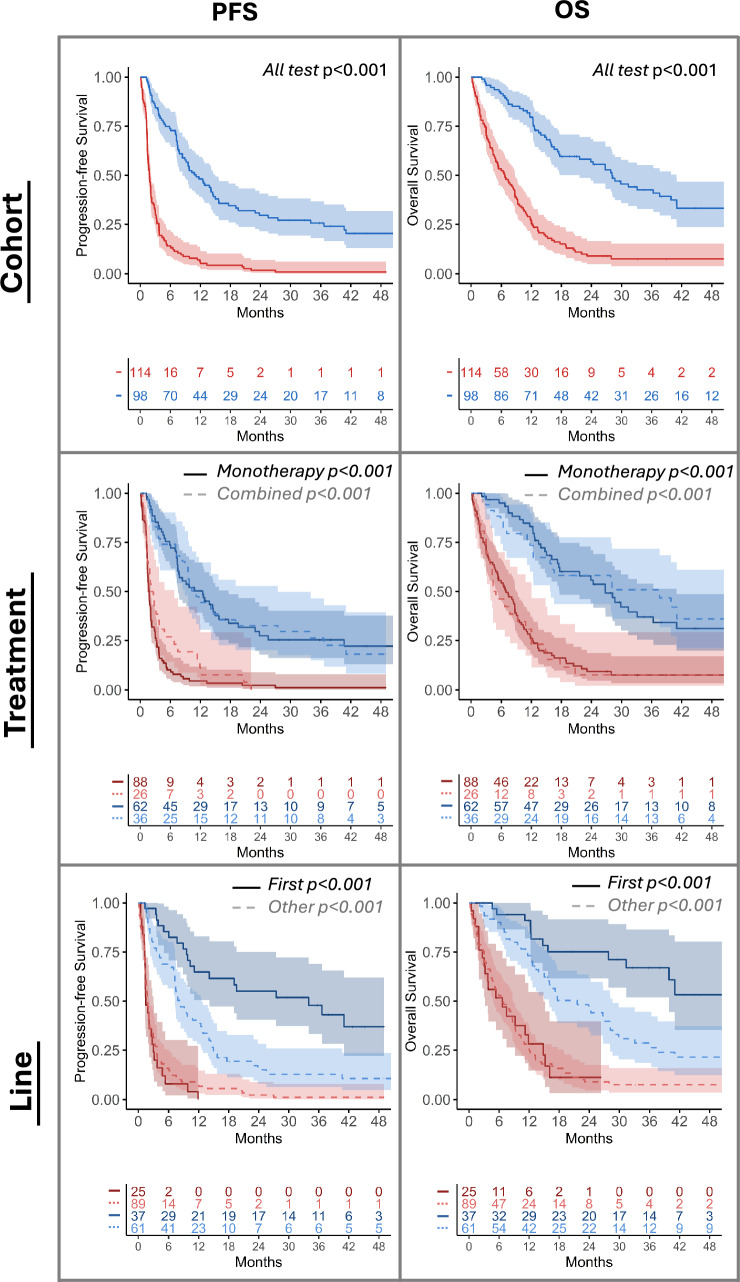
PFS and OS KM curves estimated by the multivariate longitudinal model (MV-JM) in the resultant population from merging the two testing sets, Center2 and Center3. Responders (blue) and non-responders (red) were defined by the cutoff value computed in the training set. Each row represents: (1) KM of merged testing sets, (2) monotherapy (solid line) vs. combined immunotherapy (dashed line) groups and (3) first (solid line) vs. subsequent lines (dashed line) of immunotherapy. Log-rank test p values are shown per group

PFS and OS Kaplan–Meier curves of testing patients predicted as responders and non-responders were also significantly different independently of the line of treatment, first vs subsequent lines, and the treatment option, monotherapy vs other (Fig. 4). No significant differences were found with respect to sex. Additionally, patients predicted as responders by MV-JM account for 85% and 92% of patients with an observed complete or partial response in Center2 and Center3, respectively.

## Discussion

For an automatic prediction of NSCLC response to treatment, clinical information is often used as a minor aid and the value it can have on its own over time has not yet been exploited. Our previous work has focused on the utilization of clinical information from early treatment for monitoring response, but as part of a multimodal deep-learning-based approach leveraging non-censored RWD [33]. The incorporation of censored RWD easily collected from EHRs would simplify patient eligibility and inclusion requirements would be less restrictive, bringing the algorithms closer to clinical reality. The majority of research on longitudinal RWD and JM, which allows the use of censored information, has focused on pharmacokinetics [21]. However, some studies have successfully used RWD from medical records to aid in the stratification of cancer patients and there is a growing acknowledgment of the potential value of RWD in the context of lung cancer research [34].

This study employed demographic, epidemiological, and longitudinal PBD from three clinical centers to predict the progression of patients with advanced NSCLC to immunotherapy at three distinct time points: 6, 12, and 24 months. Results from different input features, PBD sample time points, and underlying model frameworks were compared to determine which approach achieved the best performance in predicting progression to immunotherapy in a RWD setting. Previous work on JM in NSCLC treated with immunotherapy has been conducted within the context of controlled clinical trials [22]. Our study represents the first application of JM using RWD from advanced NSCLC patients to predict progression to immunotherapy.

Our results suggest that combining a stepwise selection of variables, longitudinal information and a Bayesian-based framework (MV-JM) achieved a better performance in two independent test set in terms of AUC, youden index J. Despite the higher complexity of MV-JM, 10-fold IC was lower than NLR-JM and Cox-based models. This suggests that the inclusion of additional variables and longitudinal information improves the model’s ability to explain the variability in the data more effectively. This model was also able to significantly stratify patients into high and low risk of progression ($p<0.001$) and death ($p<0.001$) according to the Kaplan–Meier curves and the long-rank test. In MV-JM, variables selected were CNS metastasis, pneumonitis, colitis, diabetes type I, LDH, LT and PLR.

CNS metastasis ($\hbox {HR}_{\text {orig}}=1.47, p<0.05$) was related to a higher progression risk and is considered a protective type of metastasis for tumor cells, given the ability of the blood–brain barrier to impair immune activity [35]. Pneumonitis ($\hbox {HR}_{\text {orig}}=2.03, p<0.05$), linked to a higher probability of progression, is considered an irAE that could aggravate pulmonary functions already compromised by cancer, while dermatitis ($\hbox {HR}_{\text {orig}}=0.62, p=0.81$) and colitis ($\hbox {HR}_{\text {orig}}=0.13, p<0.05$) could be related to an increased response and a good prognosis [36, 37]. ECOG-PS ($\hbox {HR}_{\text {orig}}=2.82, p<0.001$), associated with a higher progression risk, is related to a poor immune response [38]. Further research is required regarding to diabetes type I, associated with a better prognosis in MV-JM ($\hbox {HR}_{\text {orig}}=0.52, p=0.22$), since previous research focuses on diabetic type II [39].

LDH ($\hbox {HR}_{\text {orig}}=1.27, p<0.001$) was associated with a higher risk of progression, as a key enzyme in glycolytic metabolism in tumor cells, related to tumor evasion, metastasis, angiogenesis, and immune escape [40]. Lymphopenia confers poorer survival in immunotherapy-treated patients and an increase in LT levels post-treatment ($\hbox {HR}_{\text {orig}}=0.97, p=0.91$) have been associated with higher OS and PFS. On the contrary, platelets have been identified to express immunosuppressors associated with T cell dysfunction and PLR with immune-response impairment ($\hbox {HR}_{\text {orig}}=1.23, p=0.07$) [41–44].

The dynamics of LDH, LT and PLR during early treatment may also play an important role in patient response to immunotherapy in line with our results. According to the JM model output, intercept and slope estimated in the longitudinal submodel we could identify the expected trajectory of these features. Deviations from this linear trajectory are associated with changes in the predicted progression risk, modifying the HR originally estimated in the survival submodel (Fig. 2A) according to the dynamics of the monitored feature. MV-JM results suggest that a fast increasing LDH value, a fast decreasing LT value, and a slow decreasing PLR value from the predicted baseline values (LDH = 329.94 U/L, LT = 1543.98 /$\mu$L, PLR = 6.34) are related to a higher probability of progression. LDH has never been analyzed longitudinally for predicting immunotherapy response, in some cases paired with some temporal information, but always used at baseline [45]. On the other hand, a longitudinal NLR-based model (NLR-JM) predicts a higher probability of progression if a slow decreasing NLR value is observed from the predicted baseline value (NLR = 6.34).

Some limitations of our study comprise the retrospective nature of data, population heterogeneity, availability of longitudinal patient information, and generally short progression-free time in cohorts. Treatment heterogeneity related to immunotherapy regimes, number of lines or PDL-1-based selection criteria across centers may have influenced model performance. Particularly for Center3 MV-JM exhibited lower long-term predictive accuracy as reflected in both lower AUC values and simulated survival curves. Evolving clinical guidelines contributed to these differences. During the period when Center3 cases were treated, patients with low PD-L1 expression could receive immunotherapy as monotherapy. In contrast, current guidelines suggest combining chemotherapy with immunotherapy for such patients, highlighting the impact of temporal changes in clinical practice on the study outcomes. Despite these challenges, JM models outperformed the CPH models, likely due to their ability to account for between-subject variability through the incorporation of random effects in the longitudinal submodel. Using two independent test centers also demonstrated the superior generalizability and robustness of MV-JM. In future research we plan to explore advanced regularization methods, such as Lasso or elastic net, to address the overfitting observed in BM and DM models, as well as the integration of baseline and delta information into a single unified model. Additionally, we may include more patients with prolonged responses to predict long-term response [46] and the integration of variables studied with other relevant molecular parameters that can be monitored, such as circulating tumor DNA (ctDNA).

We have demonstrated the prognostic prediction value of monitoring peripheral blood variables and developed a decision support tool with easily collected RWD available in hospitals. Our results demonstrate that the temporal component in the model may account for changes related to the dynamics of the tumor microenvironment in three heterogenous cohorts, offsetting the lack of robustness and reliability that baseline and delta features provided on their own. Combining different variables may also lead to a more comprehensive assessment of tumor response and effective monitoring of the effects of cancer treatment compared to the use of a single biomarker such as NLR. A Bayesian joint model allows a much simpler and intuitive assessment of variable relevance for prediction compared to multimodal or deep learning approaches, which further aids the physician’s decision-making and accounts for the patient’s clinical and immunological context.

## Supplementary Information

Below is the link to the electronic supplementary material.

Supplementary file 1 (pdf 4560 KB)

## Data Availability

The data used in this study are available upon reasonable request from the corresponding author.
