# Correction: Monitoring peripheral blood data supports the prediction of immunotherapy response in advanced non‑small cell lung cancer based on real‑world data

**DOI:** 10.1007/s00262-025-04055-7

**Published:** 2025-05-27

**Authors:** Ana D. Ramos‑Guerra, Benito Farina, Jaime Rubio Pérez, Anna Vilalta‑Lacarra, Jon Zugazagoitia, Germán Peces‑Barba, Luis M. Seijo, Luis Paz‑Ares, Ignacio Gil‑Bazo, Manuel Dómine Gómez, María J. Ledesma‑Carbayo

**Affiliations:** 1 Biomedical Image Technologies, Escuela Técnica Superior de Ingenieros de Telecomunicación, Universidad Politécnica de Madrid, Madrid, Spain; 2 Centro de Investigación Biomédica en Red de Bioingeniería, Biomateriales y Nanomedicina, Instituto de Salud Carlos III, Madrid, Spain; 3 Hospital Universitario Fundación Jiménez Díaz, IIS-FJD, Madrid, Spain; 4 Memorial Sloan Kettering Cancer Center, New York, USA; 5 Department of Medical Oncology, Clínica Universidad de Navarra, Pamplona, Spain; 6 Centro de Investigación Biomédica en Red de Cáncer, Instituto de Salud Carlos III, Madrid, Spain; 7 Hospital Universitario 12 de Octubre, Madrid, Spain; 8 Centro de Investigación Biomédica en Red de Enfermedades Respiratorias, Instituto de Salud Carlos III, Madrid, Spain; 9 Department of Oncology, Hospital Vithas Vitoria, Vitoria, Spain; 10 School of Medicine, Universidad Católica de Valencia, Valencia, Spain

**Correction to: Cancer Immunology, Immunotherapy (2025) 74:120** 10.1007/s00262-025-03966-9

In the original version of this article, the affiliation details for author Ignacio Gil-Bazo were incorrectly given as

^7^ Hospital Universitario 12 de Octubre, Madrid, Spain

^9^ Department of Oncology, Hospital Vithas Vitoria, Vitoria, Spain

^10^ School of Medicine, Universidad Católica de Valencia, Valencia, Spain

but should have been

^9^ Department of Oncology, Hospital Vithas Vitoria, Vitoria, Spain

^10^ School of Medicine, Universidad Católica de Valencia, Valencia, Spain

