# Supplementary material for: Monitoring peripheral blood data supports the prediction of immunotherapy response in advanced non-small cell lung cancer based on real-world data
Source: Cancer Immunol Immunother. 2025 Feb 25;74(4):120. doi: 10.1007/s00262-025-03966-9 (PMC11861465; doi:10.1007/s00262-025-03966-9)
Supplement: Supplementary file 1 — (pdf 4560 KB) [file 262_2025_3966_MOESM1_ESM.pdf]

Supplementary material

Table S1: Summary of clinical and baseline PBD data collected in the training set Center1 and testing sets Center2 and Center3. The significancy of the difference between Center1 and Center2 and Center1 and Center3 values was computed by the Wilcoxon Mann-Whitney test for continuous variables and Pearson χ^2 and Fisher's exact test for categorical variables. *, p-value<0.05; **, p-value<0.01; ***, p-value<0.001. [a]Fisher exact test.

	**Center1**	**Center2**	***p***	**Center3**	***p***
**No. of patients**	212 (50)	137 (32)		75 (18)	
Clinical data					
**Age**			0.56		0.05
Median	66.00	64.00		68.00	
Range	36.00-88.00	32.00-89.00		39.00-87.00	
**Sex, n(%)**			0.33		** [a]
Male	143 (67)	100 (73)		64 (85)	
Female	69 (32)	37 (27)		11 (15)	
**ECOG-PFS, n(%)**			0.60		0.25
0	76 (36)	42 (31)	0.38	9 (12)	***
1	135 (64)	84 (61)	0.74	54 (72)	0.24
2	1 (1)	6 (4)	*	11 (15)	***
3	0 (0)	5 (4)	**	1 (1)	0.59
**BMI, n(%)**			0.09		***
Median	24.00	25.00		26.00	
Range	15.00-46.00	18.00-67.00		16.00-34.00	
**Diabetes, n(%)**					
No	175 (83)	124 (90)	0.06	69 (92)	0.08
Diabetes type I	5 (2)	1 (1)	0.47	0 (0)	0.41
Diabetes type II	32 (15)	12 (9)	0.12	6 (8)	0.17

Table S1 continued

	Center1	Center2	*p*	Center3	*p*
**COPD, n(%)**	66 (31)	41 (30)	0.90	21 (28)	0.72
**Pack-year**			0.14		-
Median	42.00	39.00		-	
Range	0.00-150.00	0.00-150.00		-	
**Smoking status, n(%)**					
Never smoker	18 (8)	18 (13)	0.22	3 (4)	0.31
Former smoker	124 (58)	100 (73)	**	54 (72)	0.05
Current smoker	70 (33)	19 (14)	*** [a]	18 (24)	0.19
**Surgery, n(%)**	35 (16)	20 (15)	0.74	-	-
**Steroid intake, n(%)**	86 (41)	36 (26)	**	8 (11)	*** [a]
**Antibiotic intake, n(%)**	33 (16)	33 (24)	0.06	20 (27)	0.05
**PPI, n(%)**	164 (77)	34 (25)	*** [a]	31 (41)	*** [a]
**Tumor histology, n(%)**					
Adenocarcinoma	155 (73)	112 (82)	0.08	32 (43)	*** [a]
Epidermoid	49 (23)	21 (15)	0.10	36 (48)	*** [a]
Undifferentiated	8 (4)	4 (3)	0.90	7 (9)	0.12
**PDL-1, n(%)**					
$< 1\%$	50 (24)	28 (20)	0.58	62 (83)	***
1-50%	87 (41)	81 (59)	0.51	6 (8)	***
$\geq 50\%$	75 (35)	28 (20)	**	7 (9)	***
**Driven mutations, n(%)**					
None	138 (65)	84 (61)	0.55	60 (80)	* [a]
KRAS	51 (24)	23 (17)	0.14	6 (8)	** [a]
EGFR	5 (2)	11 (8)	*	7 (9)	0.32
BRAF	5 (2)	2 (1)	0.85	1 (1)	0.95
ALK	1 (1)	3 (2)	0.34	2 (3)	0.34
ROS1	1 (1)	1 (1)	1.00	1 (1)	1.00
**No. metastatic sites, n(%)**					

Table S1 continued

	**Center1**	**Center2**	***p***	**Center3**	***p***
None	50 (24)	24 (17)	0.22	47 (63)	***
1	82 (39)	47 (34)	0.48	16 (21)	**
2	50 (24)	41 (30)	0.23	8 (11)	*
≥3	30 (14)	25 (18)	0.38	4 (5)	0.07
**Metastatic location, n(%)**					
Bone metastasis	72 (34)	39 (28)	0.34	11 (15)	** [a]
Lung metastasis	66 (31)	33 (24)	0.19	2 (3)	*** [a]
Adrenal metastasis	43 (20)	28 (20)	1.00	6 (8)	*
CNS metastasis	41 (19)	23 (17)	0.65	8 (11)	0.12
Liver metastasis	38 (18)	13 (10)	*	16 (21)	63
Nodal metastasis	28 (13)	75 (55)	*** [a]	2 (3)	** [a]
Other	32 (15)	7 (5)	** [a]	0 (0)	*** [a]
**irAEs described, n(%)**	23 (11)	15 (11)	1.00	7 (9)	1.00
Timepoint					
irAE C1-C2	4 (2)	8 (6)	0.93	3 (4)	0.26 [a]
irAE C2-C3	8 (4)	7 (5)	0.74	3 (4)	0.26 [a]
irAE C3-C5	12 (6)	6 (4)	0.78	2 (3)	0.47 [a]
Type					
Diarrhea	9 (4)	6 (4)	1.00	5 (7)	0.97
Pneumonitis	7 (3)	5 (4)	0.74	0 (0)	0.19
Thyroid dysfunction	4 (2)	1 (1)	0.67	0 (0)	0.97
Colitis	2 (1)	2 (1)	1.00	0 (0)	0.71
Dermatitis	1 (1)	0 (0)	1.00	1 (1)	1.00
Adrenal insufficiency	0 (0)	1 (1)	0.83	0 (0)	0.97
Hepatitis	0 (0)	2 (1)	0.30	1 (1)	1.00
Hypophysitis	0 (0)	1 (1)	0.80	0 (0)	1.00
Baseline PBD					
**Alb (g/dL)**			***		-
Median	4.00	4.00		-	
Range	2.70-4.90	2.20-4.30		-	

Table S1 continued

	**Center1**	**Center2**	***p***	**Center3**	***p***
**LDH (U/L)**			**		*
Median	320.00	240.00		250.00	
Range	110.00-4200.00	130.00-1500.00		150.00-840.00	
**Hb (g/dL)**			*		0.31
Median	13.00	12.00		13.00	
Range	7.50-17.00	7.40-17.00		8.50-16.00	
**PT** ($\times 10^3/\mu$L)			0.19		***
Median	300.00	290.00		270.00	
Range	45.00-860.00	59.00-690.00		120.00-720.00	
**LT** (/μL)			0.27		0.61
Median	1400.00	1300.00		1300.00	
Range	100.00-5400.00	330.00-9100.00		400.00-3400.00	
**MT** (/μL)			**		0.24
Median	600.00	710.00		700.00	
Range	200.00-6000.00	240.00-6900.00		100.00-1500.00	
**NT** (/μL)			0.26		0.92
Median	6000.00	5500.00		6000.00	
Range	900.00-29000.00	2000.00-65000.00		2600.00-22000.00	
**EOS** (/μL)			0.26		0.95
Median	100.00	130.00		100.00	
Range	0.00-1500.00	0.00-4100.00		0.00-2900.00	
**NLR**			0.77		0.53
Median	4.00	4.10		4.40	
Range	0.54-170.00	0.39-41.00		1.00-31.00	
**MLR**			***		0.17
Median	0.44	0.57		0.50	
Range	0.07-6.00	0.06-2.80		0.12-1.40	
**PLR**			0.97		0.51
Median	210.00	210.00		210.00	
Range	42.00-2000.00	23.00-930.00		47.00-1000.00	
**ALI**			0.95		0.17
Median	380.00	400.00		450.00	
Range	54.00-16000.00	28.00-4900.00		120.00-2500.00	

Table S1 continued

	**Center1**	**Center2**	***p***	**Center3**	***p***
**SII**			0.51		0.84
Median	1,200,000.00	1,200,000.00		1200000.00	
Range	62,000.00-3,4000,000.00	82000.00-11,000,000.00		220,000.00-17,000,000.00	
**PNI**			**		0.43
Median	48.00	45.00		46.00	
Range	34.00-68.00	28.00-80.00		42.00-57.00	

Table S2: Description of clinical data and baseline PBD collected in the training set Center1 and testing sets Center2 and Center3. The proportion of missing values for each variable is also included. Imputation was performed using mean substitution based on the training set values. Variables included in the multivariate joint model (MV-JM) are underlined.

		**% Imputation**		
	**Description**	**Center1**	**Center2**	**Center3**
**No. of patients**		212	137	75
**Age**	Age at baseline	0	0	0
**Sex**	Female or male	0	0	0
**<u>ECOG-PS</u>**	ECOG performance status, measures overall physical functioning (0-5)	17	7	0
**BMI**	Body-mass index: $\frac{\text{Weight (kg)}}{\text{Height (m)}^2}$	4	35†	100†
**<u>Diabetes</u>**	Diabetes type (I/II)	4	0	0
**COPD**	Chronic obstructive pulmonary disease	19	19	39†
**Pack-year**	$\frac{\text{Cigarettes per day}}{\text{Cigarettes per pack}} \times \text{Years smoked}$	7	4	100†
**Smoking status**	Smoking history: never, former, or current smoker	0	2	0
**Surgery**	Tumor resection performed before immunotherapy	0	0	100†

Table S2 continued

	Description	% Imputation		
		Center1	Center2	Center3
**Steroid intake**	Prescribed steroids during immunotherapy	0	0	0
**Antibiotic intake**	Prescribed antibiotics during immunotherapy	0	0	0
**PPI**	Prescribed proton-pump inhibitors during immunotherapy	34†	0.7	0
**Tumor histology**	Adenocarcinoma, epidermoid or undifferentiated	0.09	0	0
**PDL-1**	% PDL-1 positive tumor cells	30†	51†	0
**Driven mutations**	KRAS, EGFR, BRAF, ALK, ROS1, other or none	10	11	80†
**No. metastatic sites**	No. different organs with metastasis	0	0	0
**Metastatic location**	Organs to which the cancer has metastasized	0	0	0
**irAEs described**	Diarrhea, pneumonitus, thyroid dysfunction, colitis, dermatitis, hepatitis or hypohisitis	0	0	100†
**Alb (g/dL)**	Albumin levels	3	60†	100†
**LDH (U/L)**	Lactate dehridrogenase levels	5	8	5
**Hb (g/dL)**	Hemoglobin levels	0.4	0	5
**PT** ($\times 10^3/\mu$L)	Absolute platelet count	0.9	0	5
**LT** ($/\mu$L)	Absolute lymphocyte count	0.4	0	5
**MT** ($/\mu$L)	Absolute monocyte count	0.9	0	5
**NT** ($/\mu$L)	Absolute neutrophil count	0.4	0	5
**EOS** ($/\mu$L)	Absolute eosiophil count	1	0	5
**NLR**	$\frac{\text{NT}}{\text{LT}}$	0.4	0	5
**MLR**	$\frac{\text{MT}}{\text{LT}}$	0.9	0	5

Table S2 continued

	Description	% Imputation		
		Center1	Center2	Center3
**PLR**	$\frac{\text{PT}}{\text{LT}}$	0.9	0	5
**ALI**	$\frac{\text{ALB}}{\text{NLR}} \times \text{BMI}$	7	69†	100†
**SII**	$\text{PLR} \times \text{NT}$	0.9	0	5
**PNI**	$10 \times \text{ALB} + 0.5\%\text{LT}$	3	60†	100†

† indicates >20% missing

Table S3: Clinical data and PBD at baseline used in the best multivariate and univariate models, MV-JM and NLR-JM, both longitudinal. Notice that NLR is also used as input in NLR-BM and NLR-DM. The p-value of the difference between train (Center1) and test sets (Center2 and Center3) was computed by the Wilcoxon Mann-Whitney test and Pearson χ^2 or Fisher's exact test.

	**Center1**	**Center2**	***p***	**Center3**	***p***
MV-JM					
**Clinical data**					
ECOG-PFS, n(%)			0.60		0.25
0	76 (36)	42 (31)	0.38	9 (12)	***
1	135 (64)	84 (61)	0.74	54 (72)	0.24
2	1 (1)	6 (4)	*	11 (15)	***
3	0 (0)	5 (4)	**	1 (1)	0.59
Diabetes type I, n(%)	5 (2)	1 (1)	0.47	0 (0)	0.41
CNS metastasis, n(%)	41 (19)	23 (17)	0.65	8 (11)	0.12
Colitis, n(%)	2 (1)	2 (1)	1.00	0 (0)	0.71

Table S3 continued

	**Center1**	**Center2**	***p***	**Center3**	***p***
Dermatitis, n(%)	1 (1)	0 (0)	1.00	1 (1)	1.00
Pneumonitis, n(%)	7 (3)	5 (4)	0.74	0 (0)	0.19
**Baseline PBD**					
LDH (U/L)			**		*
Median	320.00	240.00		250.00	
Range	110.00-4200.00	130.00-1500.00		150.00-840.00	
LT(μL)			0.27		0.61
Median	1400.00	1300.00		1300.00	
Range	100.00-5400.00	330.00-9100.00		400.00-3400.00	
PLR			0.97		0.51
Median	210.00	210.00		210.00	
Range	42.00-2000.00	23.00-930.00		47.00-1000.00	
NLR-JM					
**Baseline PBD**					
NLR			0.77		0.53
Median	4.00	4.10		4.40	
Range	0.54-170.00	0.39-41.00		1.00-31.00	

*, p-value<0.05; **, p-value<0.01; ***, p-value<0.001. [a]Fisher exact test

Table S4: Summary of coefficients estimated for selected variables in the multivariate-baseline model (MV-BM) and their estimated significancy. HR_{norm} refers to the hazard ratio derived from the model, which was trained on normalized values. HR_{orig} corresponds to the hazard ratio for a one-unit increase in the original variable.

**Feature**	$\mathbf{HR}_{norm}$ **(95% CI)**	$\mathbf{HR}_{orig}$ **(95% CI)**	***p***	**RSE (%)**
ECOG-PS	4.45 (2.03-9.77)	3.21 (1.74-5.93)	***	26.88
Never smoker	1.78 (0.96-3.31)	1.78 (0.96-3.31)	0.07	54.47†
Steroid intake	0.62 (0.42-0.91)	0.62 (0.42-0.91)	*	41.03

Table S4 continued

Feature	HR_{norm} (95% CI)	HR_{orig} (95% CI)	p	RSE (%)
PPI	2.76 (1.73-4.43)	2.76 (1.73-4.43)	***	23.65
EGFR mutation	19.45 (6.24-60.68)	19.45 (6.24-60.68)	***	19.56
ROS1 mutation	8.39 (1.034-68.10)	8.39 (1.034-68.10)	*	50.23†
Other mutation	3.86 (1.727-8.62)	3.86 (1.727-8.62)	***	19.56
None metastases	21.18 (4.414-101.57)	21.18 (4.414-101.57)	***	26.20
1 metastases site	7.19 (2.90-17.80)	7.19 (2.90-17.80)	***	23.45
>3 metastases sites	0.06 (0.02-0.22)	0.06 (0.02-0.22)	***	23.90
Bone metastases	5.58 (2.39-12.99)	5.58 (2.39-12.99)	***	25.13
Lung metastases	3.84 (1.71-8.64)	3.84 (1.71-8.64)	**	30.74
Adrenal metastases	8.77 (3.69-20.87)	8.77 (3.69-20.87)	***	20.37
CNS metastases	5.49 (2.46-12.24)	5.49 (2.46-12.24)	***	24.00
Liver metastases	10.91 (4.47-26.61)	10.91 (4.47-26.61)	***	19.03
Nodal metastases	3.78 (1.46-9.77)	3.78 (1.46-9.77)	**	36.44
Oth metastases	2.02 (1.22-3.36)	2.02 (1.22-3.36)	**	36.56
Any irAE	6.53 (2.02-21.11)	6.53 (2.02-21.11)	**	31.91
irAE t1-t3	0.20 (0.05-0.74)	0.20 (0.05-0.74)	*	41.48

Table S4 continued

**Feature**	**HR**$_{norm}$ **(95% CI)**	**HR**$_{orig}$ **(95% CI)**	p	**RSE (%)**
Pneumonitis	4.02 (1.41-11.47)	4.02 (1.41-11.47)	**	38.40
Colitis	0.22 (0.02-2.03)	0.22 (0.02-2.03)	0.18	75.13†
ALB	0.07 (0.02-0.21)	0.06 (0.02-0.20)	***	21.30
LDH	49.17 (6.67-362.40)	1.24 (1.11-1.38)	***	26.16
Hb	5.18 (1.68-15.96)	2.92 (1.40-6.09)	**	34.89
ALI	0.08 (0.01-0.80)	0.92 (0.86-0.99)	*	46.45

* p-value<0.05, ** p-value<0.01, *** p-value<0.001. † RSE>50%

Table S5: Summary of coefficients estimated for selected variables in the NLR-baseline model (NLR-BM) and their estimated significancy.HR$_{norm}$ refers to the hazard ratio derived from the model, which was trained on normalized values. HR$_{orig}$ corresponds to the hazard ratio for a one-unit increase in the original variable.

**Feature**	**HR**$_{norm}$ **(95% CI)**	**HR**$_{orig}$ **(95% CI)**	p	**RSE (%)**
NLR	6.61 (1.46-29.92)	1.07 (1.01-1.14)	*	40.76

* p-value<0.05, ** p-value<0.01, *** p-value<0.001. † RSE>50%

Table S6: Summary of coefficients estimated for selected variables in the multivariate-delta model (MV-DM) and their estimated significancy. For monitored features, Δfeature = feature_{C2}- feature_{C1}, where C1 and C2 are the first and second cycle of immunotherapy. HR_{norm} refers to the hazard ratio derived from the model, which was trained on normalized values. HR_{orig} corresponds to the hazard ratio for a one-unit increase in the original variable.

**Feature**	$\textbf{HR}_{norm}$ **(95% CI)**	$\textbf{HR}_{orig}$ **(95% CI)**	***p***	**RSE (%)**
ECOG-PS	3.47 (0.80-14.96)	2.65 (0.84-8.31)	0.10	59.95†
COPD	2.15 (1.17-3.94)	2.15 (1.17-3.94)	*	40.41
Former smoker	0.45 (0.24-0.83)	0.45 (0.24-0.83)	*	39.05
Adenocarcinoma	5.12 (2.41-10.87)	5.12 (2.41-10.87)	***	23.52
PDL1	4.08 (1.66-10.06)	4.08 (1.66-10.06)	**	32.70
Other mutation	30.75 (5.13-184.30)	30.75 (5.13-184.30)	***	26.67
None metastasis	278.80 (18.28-4,253.00)	278.80 (18.28-4,253.00)	***	24.69
1 metastatic site	5.26 (1.13-24.57)	5.26 (1.13-24.57)	*	47.35
>3 metastases sites	0.01 (1.88×10^{-3}-0.10)	0.01 (1.88×10^{-3}-0.10)	***	23.48
Bone metastases	19.52 (4.71-80.90)	19.52 (4.71-80.90)	***	24.42
Lung metastases	36.05 (8.35-155.60)	36.05 (8.35-155.60)	***	20.81
Adrenal metastases	18.39 (4.72-71.71)	18.39 (4.72-71.71)	***	23.84
CNS metastases	82.47 (16.68-407.80)	82.47 (16.68-407.80)	***	18.48
Liver metastases	50.03 (8.96-279.40)	50.03 (8.96-279.40)	***	22.43

Table S6 continued

**Feature**	**HR$_{norm}$ (95% CI)**	**HR$_{orig}$ (95% CI)**	p	**RSE (%)**
Nodal metastases	15.24 (2.69-86.25)	15.24 (2.69-86.25)	**	32.47
Other metastases	7.09 (2.75-18.30)	7.09 (2.75-18.30)	***	24.69
irAE t1-t3	0.36 (0.11-1.21)	0.36 (0.11-1.21)	0.10	60.48†
Pneumonitis	142.00 (24.34-828.80)	142.00 (24.34-828.80)	***	18.16
ΔALB	1.18×10^{-7} (8.65×10^{-13}-0.02)	1.74×10^{-6} (9.34×10^{-11}-0.04)	**	37.81
ΔLDH	76.23 (6.38-910.50)	3.72 (1.75-7.90)	***	29.20
ΔLT	1.19×10^{-4} (4.56×10^{-8}-0.31)	0.02 (9.50×10^{-4}-0.62)	*	44.44
ΔNLR	4.61×10^{-24} (2.76×10^{-41}-7.68×10^{-7})	3.08×10^{-18} (3.67×10^{-31}-2.58×10^{-5})	**	37.6
ΔMLR	92.68 (2.15-3,993.00)	13.61 (1.55-119.08)	*	42.39
ΔPLR	0.01 (7.94×10^{-5}-0.57)	0.16 (0.02-0.80)	*	45.32
ΔPNI	1.81×10^{7} (25.49-1.29×10^{13})	1673.04 (4.21-6.65×10^{5})	*	41.14
ΔALI	3.79×10^{25} (1.02×10^{9}-1.41×10^{42})	1.39×10^{21} (2.75×10^{7}-6.43×10^{34})	**	33.05

* p-value<0.05, ** p-value<0.01, *** p-value<0.001. † RSE>50%

Table S7: Summary of coefficients estimated for selected variables in the NLR-delta model (NLR-DM) and their estimated significancy. ΔNLR = NLR_{C2}- NLR_{C1}, where C1 and C2 are the first and second cycle of immunotherapy.HR_{norm} refers to the hazard ratio derived from the model, which was trained on normalized values. HR_{orig} corresponds to the hazard ratio for a one-unit increase in the original variable.

**Feature**	**HR_{norm} (95% CI)**	**HR_{orig} (95% CI)**	***p***	**RSE (%)**
ΔNLR	0.25 (0.01-4.32)	0.35 (0.03-3.00)	0.34	104.31†

* p-value<0.05, ** p-value<0.01, *** p-value<0.001. † RSE>50%

Table S8: Summary of the multivariate-longitudinal model (MV-JM) poterior mean of hazard ratios (HR) and significancy associated to each variable in the survival and longitudinal submodel, trained on Center1, N=212. In the longitudinal submodel, the intercept models the expected value at baseline, slope the rate change of the temporal variable and σ the variability in measurements not explained by the model.HR_{norm} refers to the hazard ratio derived from the model, which was trained on normalized values. HR_{orig} corresponds to the hazard ratio for a one-unit increase in the original variable.

**Survival outcome**	**Feature**	**HR_{norm} (95% CI)**	**HR_{orig} (95% CI)**	***p***	**RSE (%)**
	Diabetes type I	0.52 (0.16-1.46)	0.52 (0.16-1.46)	0.22	3.66
	CNS metastases	1.47 (1.03-2.01)	1.47 (1.03-2.01)	*	0.93
	Pneumonitis	2.03 (1.05-3.80)	2.03 (1.05-3.80)	*	1.62
	Dermatitis	0.62 (0.03-4.88)	0.62 (0.03-4.88)	0.81	10.37
	Colitis	0.13 (0.01-0.80)	0.13 (0.01-0.80)	*	1.66
	ECOG-PS	3.77 (2.34-5.89)	2.82 (1.94-3.99)	***	0.54
	LDH	78.44 (17.83-285.49)	1.27 (1.17-1.36)	***	0.86

Table S8 continued

**Survival outcome**	**Feature**	**HR_{norm} (95% CI)**	**HR_{orig} (95% CI)**	***p***	**RSE (%)**
	LT	0.89 (0.18-4.14)	0.97 (0.63-1.47)	0.91	21.17
	PLR	5.81 (0.86-43.96)	1.23 (0.98-1.56)	0.07	1.48
**Longitudinal outcome**					
	LDH				
	exp(Intercept)	1.06 (1.05-1.06)	1.003 (1.002-1.003)	***	0.19
	exp(slope)	1.01 (0.99-1.03)	1.0005 (0.9994-1.0016)	0.36	3.88
	exp(σ)	1.02 (1.01-1.02)	1.0011 (1.0005-1.0011)	***	0.15
	LT				
	exp(Intercept)	1.31 (1.29-1.34)	1.07 (1.07-1.08)	***	0.11
	exp(slope)	0.99 (0.99-1.01)	0.997 (0.997-1.003)	0.99	268.65†
	exp(σ)	1.12 (1.11-1.12)	1.031 (1.029-1.031)	***	0.10
	PLR				
	exp(Intercept)	1.12 (1.11-1.14)	1.013 (1.012-1.016)	***	0.16
	exp(slope)	0.99 (0.99-1.00)	0.99 (0.99-1.00)	0.07	1.10
	exp(σ)	1.05 (1.05-1.06)	1.006 (1.006-1.007)	***	0.11

* p-value<0.05, ** p-value<0.01, *** p-value<0.001. † RSE>50%

Table S9: Summary of longitudinal NLR-based (NLR-JM) poterior mean of hazard ratios (HR) and significancy associated to each variable, trained on Center1, N=212. In the longitudinal submodel, the intercept models the expected value at baseline, slope the rate change of the temporal variable and σ the variability in measurements not explained by the model.HR_{norm} refers to the hazard ratio derived from the model, which was trained on normalized values. HR_{orig} corresponds to the hazard ratio for a one-unit increase in the original variable.

**Survival outcome**	**Feature**	$\mathbf{HR}_{norm}$ **(95% CI)**	$\mathbf{HR}_{orig}$ **(95% CI)**	$\boldsymbol{p}$	**RSE (%)**
	NLR	4.68 (0.52-105.63)	1.06 (0.97-1.19)	0.19	2.90
**Longitudinal outcome**					
	NLR				
	exp(Intercept)	1.03 (1.03-1.04)	1.001 (1.001-1.002)	***	0.27
	exp(slope)	0.99 (0.98-0.99)	0.9996 (0.9996-0.9992)	***	0.73
	exp(σ)	1.03 (1.02-1.03)	1.0011 (1.0008-1.0011)	***	0.11

* p-value<0.05, ** p-value<0.01, *** p-value<0.001. † RSE>50%

Table S10: Overview of optimal models. Model variables, longitudinal samples used (cycles), and the 10-fold information criteria (IC) and standard error (SE) of each model are detailed. The models are classified as follows: MV (multivariate) or NLR (neutrophil-to-lymphocyte ratio) and BM (baseline model, single time point), DM (delta model, two time points), or JM (joint longitudinal model, four time points). Lowest 10-foldIC is presented in bold for each model (BM, DM or JM) when comparing MV and NLR sets of features.

**Model**	**cycles**	**Variables**	**10-fold IC (SE)**
**MV-BM**	C1	ECOG-PS, Never smoker, Steroid intake, PPI, EGFR, ROS1, Other and None mutation, 1 and >3 metastases site, Bone, Lung, Adrenal, CNS, Liver, Nodal and Other metastases, any irAE and irAE t1-t3, Pneumonitis, Colitis, ALB, LDH, Hb, ALI	**1231.01 (2.32)**
**NLR-BM**	C1	NLR	1284.95 (1.27)
**MV-DM**	C1,C2	ECOG-PS,COPD,Former smoker, Adenocarcinoma, PDL-1, Other and None mutations, 1 and >3 metastases sites, Bone, Lung, Adrenal, CNS, Liver, Nodal and Other metastases, irAE t1-t3, Pneumonitis, ALB, LDH, LT, NLR, MLR, PLR, PNI, ALI	**502.11 (6.03)**
**NLR-DM**	C1,C2	NLR	541.38 (5.32)
**MV-JM**	C1,C2,C3,C5	Diabetes type I, CNS metastases, Pneumonitis, Dermatitis, Colitis, ECOG-PS, LDH, LT, PLR	**-1630.76 (150.30)**
**NLR-JM**	C1,C2,C3,C5	NLR	-1317.21 (47.50)

Table S11: Summary of AUC, sensitivity (SENS) and specifity (SPEC) and SD for each cohort of patients and multivariate baseline (MV-BM), delta (MV-DM) and longitudinal (MV-JM) models.

			**MV-BM**			**MV-DM**			**MV-JM**	
	Mo.	*AUC*	*SENS*	*SPEC*	*AUC*	*SENS*	*SPEC*	*AUC*	*SENS*	*SPEC*
**Training** **Center1**	*6*	0.817 (0.029)	0.636 (0.046)	0.890 (0.031)	0.875 (0.035)	0.732 (0.064)	0.912 (0.038)	0.797 (0.032)	0.664 (0.048)	0.790 (0.039)
	12	0.777 (0.034)	0.509 (0.042)	0.922 (0.037)	0.843 (0.039)	0.586 (0.060)	1.000 (0.000)	0.753 (0.039)	0.547 (0.044)	0.824 (0.052)
	24	0.774 (0.042)	0.465 (0.040)	1.000 (0.000)	0.897 (0.034)	0.482 (0.058)	1.000 (0.000)	0.760 (0.058)	0.501 (0.042)	0.810 (0.087)
**Testing** **Center2**	*6*	0.626 (0.048)	0.419 (0.057)	0.897 (0.041)	0.492 (0.054)	0.357 (0.062)	0.630 (0.068)	0.870 (0.031)	0.788 (0.047)	0.810 (0.051)
	12	0.582 (0.053)	0.344 (0.048)	0.882 (0.055)	0.433 (0.059)	0.323 (0.053)	0.581 (0.091)	0.804 (0.040)	0.659 (0.047)	0.853 (0.062)
	24	0.626 (0.055)	0.330 (0.044)	0.955 (0.047)	0.528 (0.068)	0.375 (0.052)	0.714 (0.102)	0.827 (0039)	0.626 (0.044)	0.955 (0.046)
**Testing** **Center3**	*6*	0.558 (0.073)	0.340 (0.070)	0.750 (0.085)	0.533 (0.068)	0.667 (0.069)	0.393 (0.092)	0.824 (0.060)	0.809 (0.059)	0.821 (0.075)
	12	0.474 (0.080)	0.310 (0.064)	0.688 (0.118)	0.574 (0.081)	0.642 (0.066)	0.375 (0.123)	0.822 (0.064)	0.724 (0.059)	0.938 (0.063)
	24	0.438 (0.215)	0.297 (0.056)	0.500 (0.292)	0.480 (0.189)	0.626 (0.062)	0.250 (0.265)	0.667 (0.206)	0.615 (0.059)	0.750 (0.244)
**All**	*6*	0.699 (0.027)	0.501 (0.034)	0.871 (0.033)	0.645 (0.033)	0.546 (0.042)	0.705 (0.041)	0.832 (0.021)	0.736 (0.029)	0.801 (0.029)
	12	0.657 (0.030)	0.415 (0.029)	0.871 (0.029)	0.609 (0.038)	0.483 (0.034)	0.707 (0.036)	0.787 (0.025)	0.619 (0.028)	0.851 (0.028)
	24	0.685 (0.036)	0.389 (0.027)	0.936 (0.027)	0.681 (0.045)	0.475 (0.034)	0.795 (0.033)	0.792 (0.034)	0.566 (0.027)	0.872 (0.027)

Table S12: Summary of AUC, sensitivity and specifity and SD for each cohort of patients in NLR baseline (NLR-BM), delta (NLR-DM) and longitudinal (NLR-JM) models. In bold, AUC values for the JM model variations.

			**NLR-BM**			**NLR-DM**			**NLR-JM**	
	Mo.	*AUC*	*SENS*	*SPEC*	*AUC*	*SENS*	*SPEC*	*AUC*	*SENS*	*SPEC*
**Training** **Center1**	*6*	0.605 (0.039)	0.322 (0.046)	0.780 (0.041)	0.460 (0.061)	0.647 (0.072)	0.263 (0.059)	0.722 (0.038)	0.920 (0.027)	0.450 (0.051)
	12	0.541 (0.047)	0.288 (0.037)	0.745 (0.061)	0.428 (0.064)	0.668 (0.060)	0.214 (0.079)	0.685 (0.046)	0.844 (0.030)	0.510 (0.070)
	24	0.549 (0.062)	0.283 (0.036)	0.857 (0.079)	0.489 (0.087)	0.708 (0.054)	0.286 (0.128)	0.705 (0.058)	0.787 (0.034)	0.524 (0.115)
**Testing** **Center2**	*6*	0.610 (0.049)	0.355 (0.057)	0.759 (0.057)	0.307 (0.052)	0.500 (0.070)	0.204 (0.052)	0.857 (0.033)	0.933 (0.029)	0.379 (0.063)
	12	0.584 (0.060)	0.325 (0.046)	0.735 (0.076)	0.376 (0.059)	0.600 (0.056)	0.226 (0.076)	0.776 (0.043)	0.875 (0.034)	0.412 (0.085)
	24	0.507 (0.071)	0.295 (0.042)	0.636 (0.103)	0.335 (0.067)	0.628 (0.052)	0.238 (0.094)	0.792 (0.048)	0.854 (0.034)	0.500 (0.108)
**Testing** **Center3**	*6*	0.529 (0.067)	0.298 (0.068)	0.786 (0.080)	0.452 (0.071)	0.643 (0.072)	0.250 (0.083)	0.842 (0.046)	0.891 (0.046)	0.500 (0.096)
	12	0.494 (0.073)	0.276 (0.058)	0.750 (0.110)	0.402 (0.073)	0.660 (0.063)	0.188 (0.099)	0.823 (0.053)	0.845 (0.048)	0.563 (0.125)
	24	0.506 (0.207)	0.250 (0.052)	0.500 (0.292)	0.383 (0.151)	0.679 (0.059)	0.250 (0.241)	0.682 (0.135)	0.798 (0.050)	0.250 (0.252)
**All**	*6*	0.597 (0.028)	0.328 (0.032)	0.774 (0.032)	0.399 (0.033)	0.588 (0.040)	0.237 (0.040)	0.777 (0.023)	0.919 (0.019)	0.435 (0.019)
	12	0.551 (0.033)	0.299 (0.027)	0.743 (0.026)	0.401 (0.039)	0.641 (0.036)	0.231 (0.034)	0.733 (0.028)	0.855 (0.021)	0.485 (0.021)
	24	0.529 (0.046)	0.280 (0.026)	0.723 (0.024)	0.396 (0.050)	0.669 (0.031)	0.256 (0.031)	0.730 (0.036)	0.812 (0.023)	0.489 (0.022)

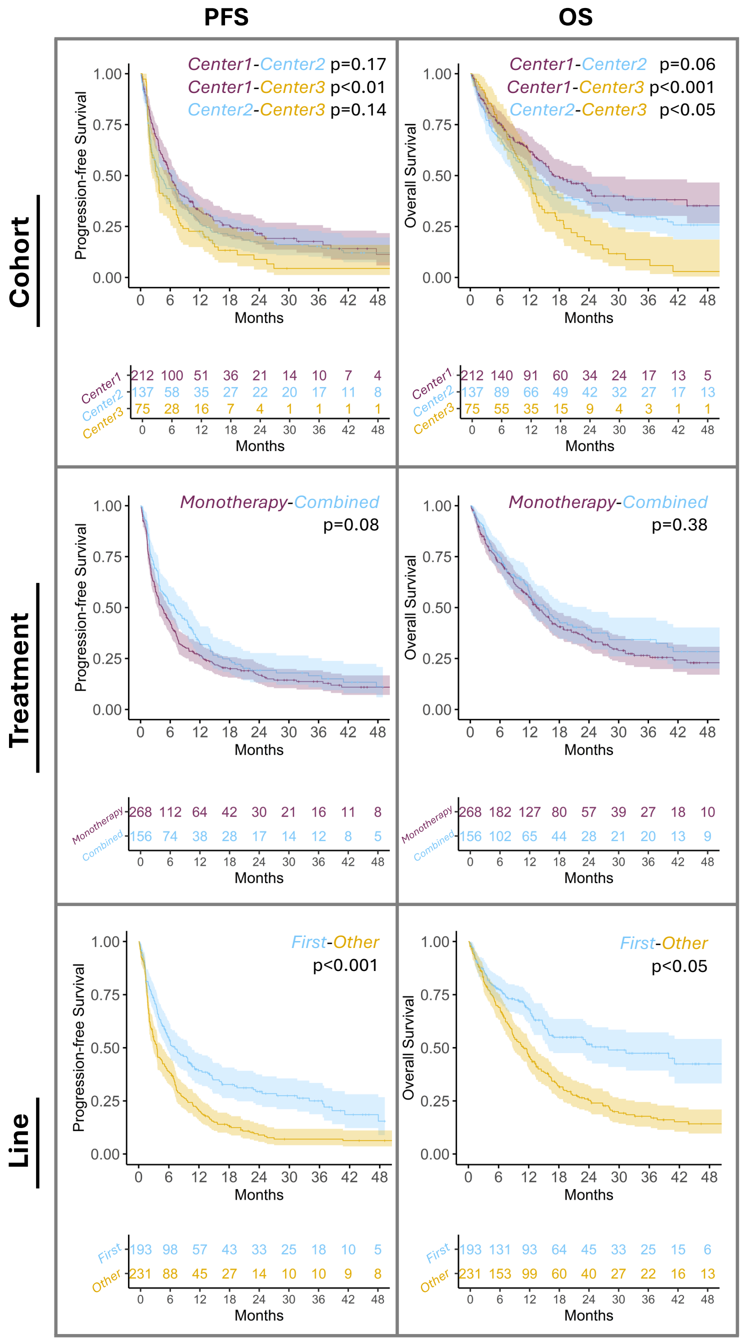

Fig. S1: Kaplan-Meier curves and log-rank test p-values for progression-free survival (left) and overall survival (right) considering the patients from all institutions. The curves in each row represent: (1) study cohorts Center1, training set, and Center2 and Center3, testing sets; (2) treatment as monotherapy vs combined immunotherapy; and (3) line of treatment as first-line vs subsequent lines.

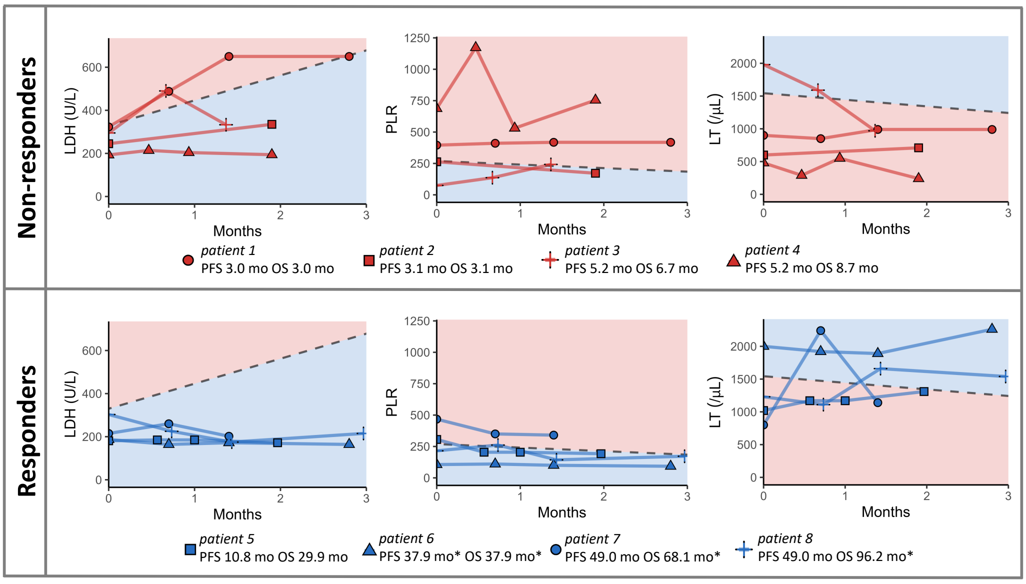

Fig. S2: Trajectories of LDH, PLR and LT monitored during the first three months of immunotherapy of four non-responders (patients 1-4) and four responders (patients 5-8) from the testing cohorts with the highest confidence of prediction in the multivariate longitudinal model (MV-JM), confidence > 0.7. Feature values are represented on the original scale, not normalized. The modeled effect on progression of a feature is represented as red (non-responders) and blue (responders) shaded areas and is based on model hazard ratios. Deviations from the expected trajectory (gray dashed line) mean a higher or lower estimated progression risk according to model. Progression-free and overall survival of each patient is expressed in months. * Censored cases.

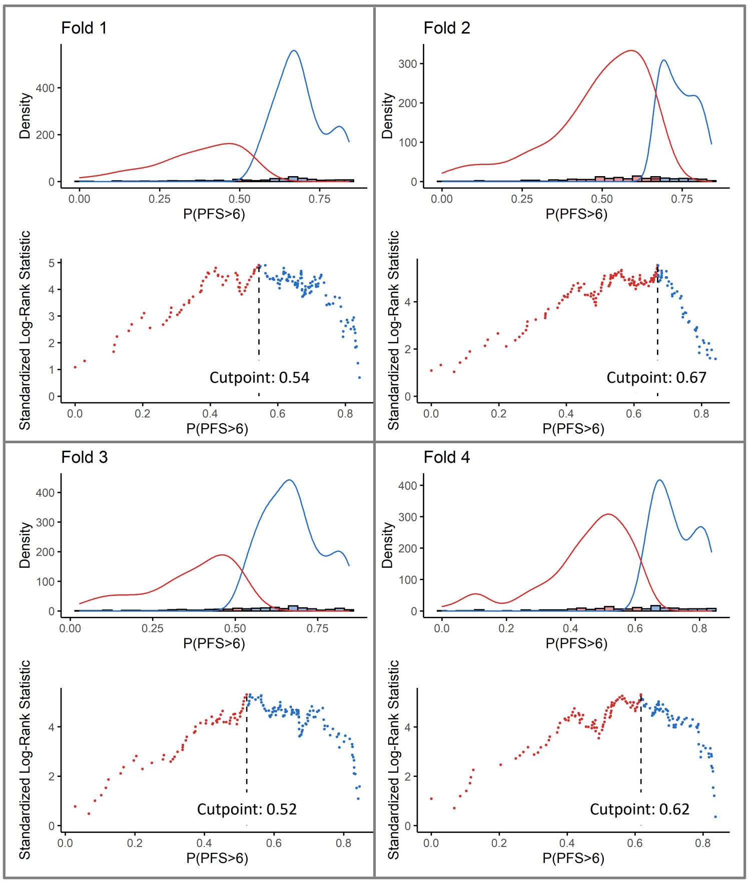

Fig. S3: Distribution of the probablity of progression after 6 months P(PFS>6) computed by MV-JM in the training set Center1 and the standarized log-rank statistic. The vertical dashed line represents the optimal cutoff point identified in the training set for each fold after 4-fold cross-validation. The average cutoff point (0.59) was applied to classify patients into high- (red) and low-risk (blue) groups.

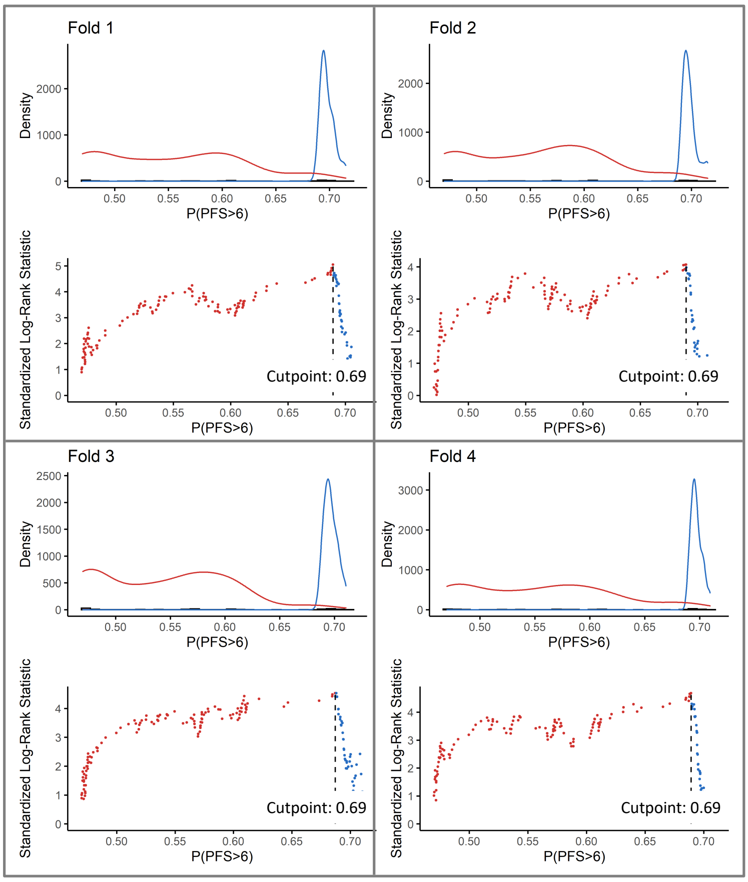

Fig. S4: Distribution of the probablity of progression after 6 months P(PFS>6) computed by NLR-JM in the training set Center1 and the standarized log-rank statistic. The vertical dashed line represents the optimal cutoff point identified in the training for each fold after 4-fold cross-validation. The average cutoff point (0.69) was applied to classify patients into high- (red) and low-risk (blue) groups.

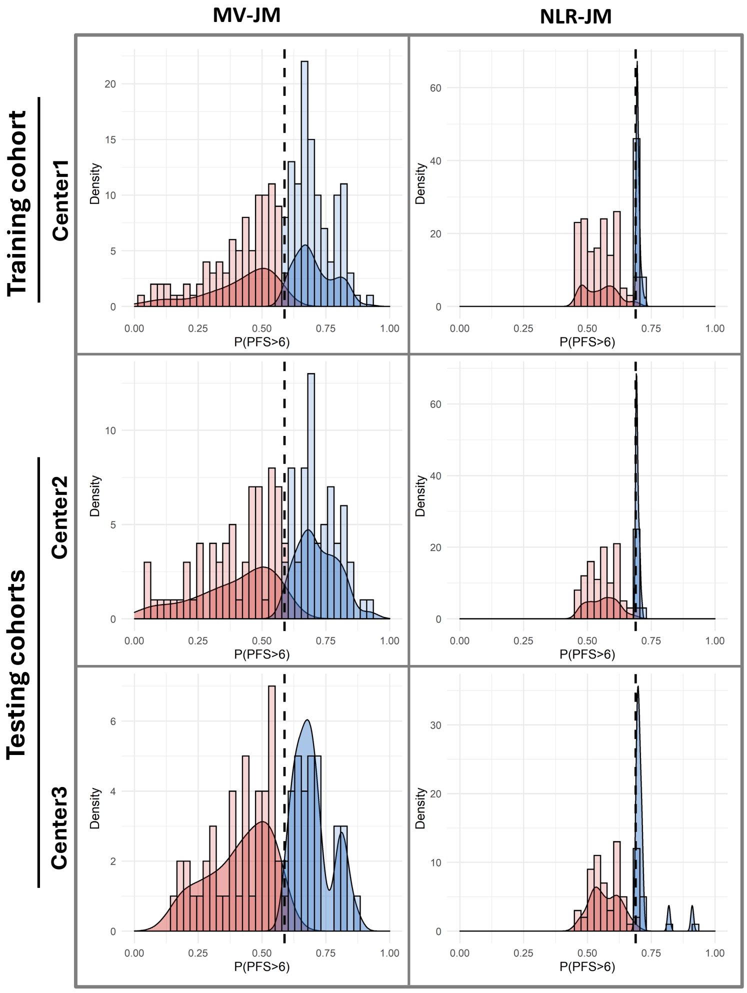

Fig. S5: Distribution of the probability of progression after 6 months computed by MV-JM and NLR-JM in the training set Center1 and the testing sets Center2 and Center3. The vertical dashed lines indicate the optimal cutoff points identified in the training set, derived by averaging the cutoff values from 4-fold cross-validation. The distribution of high-risk patients is in red and of low risk in blue. The average cutoff points (0.59 for MV-JM and 0.69 for NLR-JM) were applied uniformly to classify patients into high- and low-risk groups.

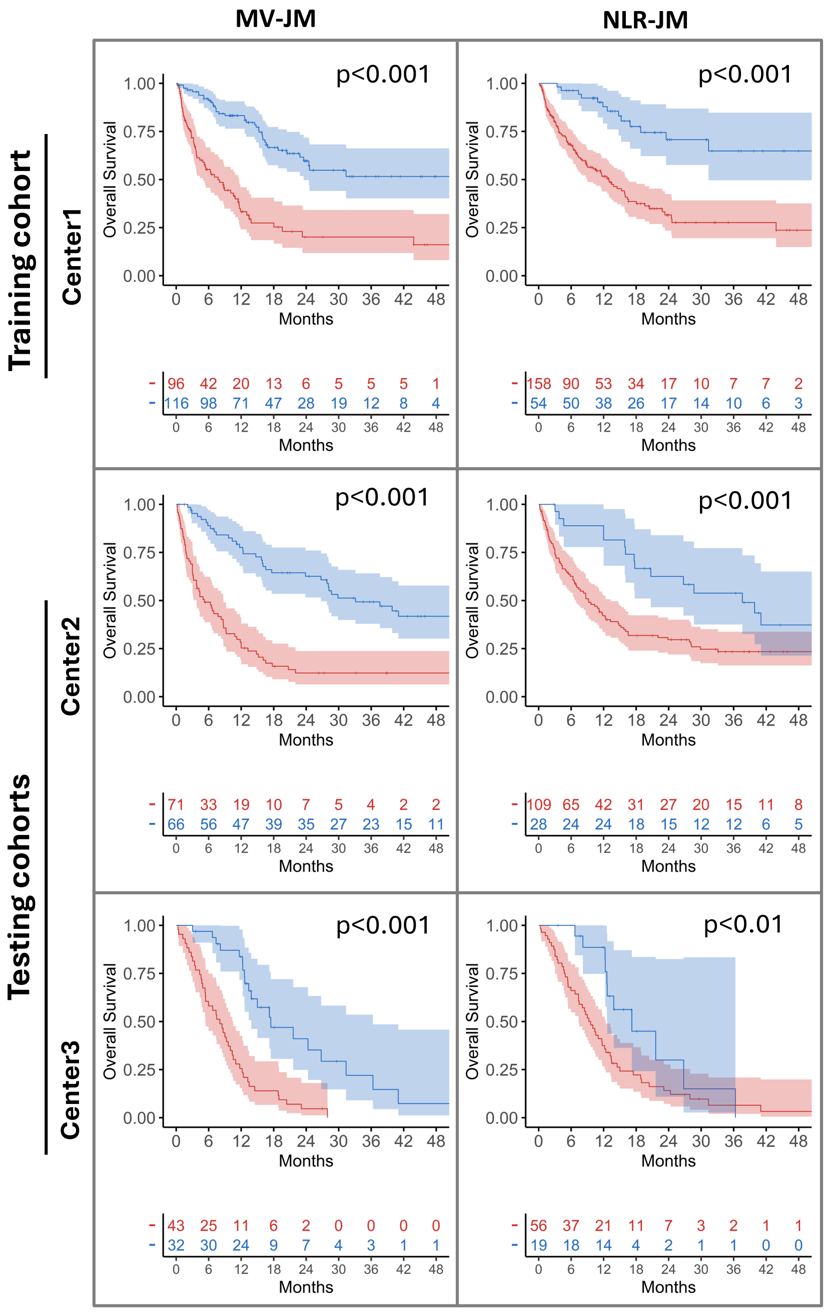

Fig. S6: OS Kaplan-Meier curves estimated by the MV-JM and NLR-JM models predicting high (red) or low risk of progression (blue) in Center1 training set and Center2 and Center3 testing sets. Plotted groups were generated by the corresponding cutoff value for each model, computed in the training set and applied to both testing sets. Log-rank test p-value is shown.
